# [Retracted] Role of long non‑coding RNA MALAT1 in chronic obstructive pulmonary disease

**DOI:** 10.3892/etm.2024.12740

**Published:** 2024-10-07

**Authors:** Tian-Jun Hu, Hong-Bo Huang, Hai-Bo Shen, Wei Chen, Zhen-Hua Yang

Exp Ther Med 20:2691–2697, 2020; DOI: 10.3892/etm.2020.8996

Following the publication of this paper, it was drawn to the Editor’s attention by a concerned reader that the immunofluorescence assay data in Fig. 3 on p. 2694 and the control western blot assay data shown in Fig. 4A on p. 2695 were strikingly similar to data appearing in different form in another article written by different authors at different research institutes that had already been accepted for publication in the journal *Archives of Medical Science* prior to the receipt of this paper at *Experimental and Therapeutic Medicine.*

In view of the fact that the abovementioned data had already apparently been accepted for publication in another journal before this paper was received at *Experimental and Therapeutic Medicine*, the Editor has decided that it should be retracted from the Journal. The authors were asked for an explanation to account for these concerns, but the Editorial Office did not receive a reply. The Editor apologizes to the readership for any inconvenience caused.

